# Population genetics of *Leishmania (Leishmania) major* DNA isolated from cutaneous leishmaniasis patients in Pakistan based on multilocus microsatellite typing

**DOI:** 10.1186/1756-3305-7-332

**Published:** 2014-07-16

**Authors:** Mohammad Zahangir Alam, Abdul Manan Bhutto, Farooq Rahman Soomro, Javed Hussain Baloch, Ryo Nakao, Hirotomo Kato, Gabriele Schönian, Hiroshi Uezato, Yoshihisa Hashiguchi, Ken Katakura

**Affiliations:** 1 Department of Disease Control, Laboratory of Parasitology, Graduate School of Veterinary Medicine, Hokkaido University, Kita 18 Nishi 9, Kita-ku, Sapporo 060-0818, Japan; 2 Department of Parasitology, Faculty of Veterinary Science, Bangladesh Agricultural University, Mymensingh 2202, Bangladesh; 3 Department of Dermatology, Shaheed Mohtarma Benazir Bhutto Medical University, Larkana, Pakistan; 4 Leprosy Centre, Larkana, Pakistan; 5 Unit of Risk Analysis and Management, Research Center for Zoonosis Control, Hokkaido University, Sapporo 001-0020, Japan; 6 Institut für Mikrobiologie und Hygiene, Charité Universitätsmedizin Berlin, Berlin, Germany; 7 Division of Dermatology, Department of Organ-oriented Medicine, School of Medicine, University of the Ryukyus, Okinawa 903-0215, Japan; 8 Department of Parasitology, Kochi Medical School, Kochi University, Kochi 783-8505, Japan; 9 Centro de Biomedicina, Universidad Central del Ecuador and Prometeo Project, SENESCYT, Quito, Ecuador

**Keywords:** *Leishmania (Leishmania) major*, Microsatellite typing, Population genetics, Pakistan

## Abstract

**Background:**

Cutaneous leishmaniasis (CL) is a major and fast increasing public health problem, both among the local Pakistani populations and the Afghan refugees in camps. *Leishmania (Leishmania) major* is one of the etiological agents responsible for CL in Pakistan*.* Genetic variability and population structure have been investigated for 66 DNA samples of *L. (L.) major* isolated from skin biopsy of CL patients.

**Methods:**

Multilocus microsatellite typing (MLMT), employing 10 independent genetic markers specific to *L. (L.) major*, was used to investigate the genetic polymorphisms and population structures of Pakistani *L. (L.) major* DNA isolated from CL human cases. Their microsatellite profiles were compared to those of 130 previously typed strains of *L. (L.) major* from various geographical localities.

**Results:**

All the markers were polymorphic and fifty-one MLMT profiles were recognized among the 66 *L. (L.) major* DNA samples. The data displayed significant microsatellite polymorphisms with rare allelic heterozygosities. A Bayesian model-based approach and phylogenetic analysis inferred two *L. (L.) major* populations in Pakistan. Thirty-four samples belonged to one population and the remaining 32 *L. (L.) major* samples grouped together into another population. The two Pakistani *L. (L.) major* populations formed separate clusters, which differ genetically from the populations of *L. (L.) major* from Central Asia, Iran, Middle East and Africa.

**Conclusions:**

The considerable genetic variability of *L. (L.) major* might be related to the existence of different species of sand fly and/or rodent reservoir host in Sindh province, Pakistan. A comprehensive study of the epidemiology of CL including the situation or spreading of reservoirs and sand fly vectors in these foci is, therefore, warranted.

## Background

Leishmaniases are a group of parasitic diseases caused by obligatory, intracellular, protozoan parasites of the genus *Leishmania.* Cutaneous leishmaniasis (CL) is more widely distributed, with about one-third of cases occurring in each of three epidemiological regions, the Americas, the Mediterranean basin, and western Asia from the Middle East to Central Asia [1]. Pakistan, a tropical and subtropical country located in the northwest of South Asia, is highly endemic for the leishmaniases. CL is a major and fast increasing public health problem, both among the local Pakistani populations and the Afghan refugees in camps. Its extensive spread has been associated with mass migration, from endemic to non-endemic areas and *vice versa*, and with Afghan refugees from areas where CL is highly endemic [2]. Recently, more than 1200 cases of CL were identified in the lowland of Sindh province, the southern part of Pakistan [3]. Two types of CL, anthroponotic (ACL) and zoonotic (ZCL) are prevalent in Pakistan. Zoonotic CL caused by *Leishmania (Leishmania) major* mainly occurs in rural and semi-urban areas of Balochistan and neighboring Punjab and Sindh provinces. Clinically, the disease has been associated with “moist or wet-type” lesions, but unusual clinical forms have also been reported [4,5].

The parasites from the lowland areas of Sindh province were assigned to *L. (L.) major* by multilocus enzyme electrophoresis (MLEE) and intra-specific polymorphisms were reported among these *L. (L.) major* isolates [6]. Typing of *L. (L.) major* parasites from Pakistan by using PCR-based methods targeting nuclear multicopy sequences or antigen-coding genes, followed by subsequent search for polymorphism by sequencing showed little genetic variation within this species [7]. For population genetic studies and differentiation of closely related parasites, markers of higher discriminatory power are needed. Multilocus microsatellite typing (MLMT) has become an increasingly important tool for molecular typing and population genetic studies in different species of the genus *Leishmania* and data obtained by MLMT are highly informative in an eco-geographical context [8-12]. MLMT has the advantage of providing reproducible results that can be stored as databases for sharing among different laboratories, including its use for predicting evolutionary origin of the *Leishmania* parasites [11,13]. Recently, microsatellite markers were used to infer the population structure of *L. (L.) major* on a global scale [12] and on a country-wide scale in Iran [14].

In the present study, we used a panel of previously described microsatellite markers [12] to investigate the genetic variation and population structure of Pakistani *L. (L.) major* isolates, and to compare them with strains from other endemic foci in different geographical areas.

## Methods

### *Leishmania* DNA

**Figure 1:**
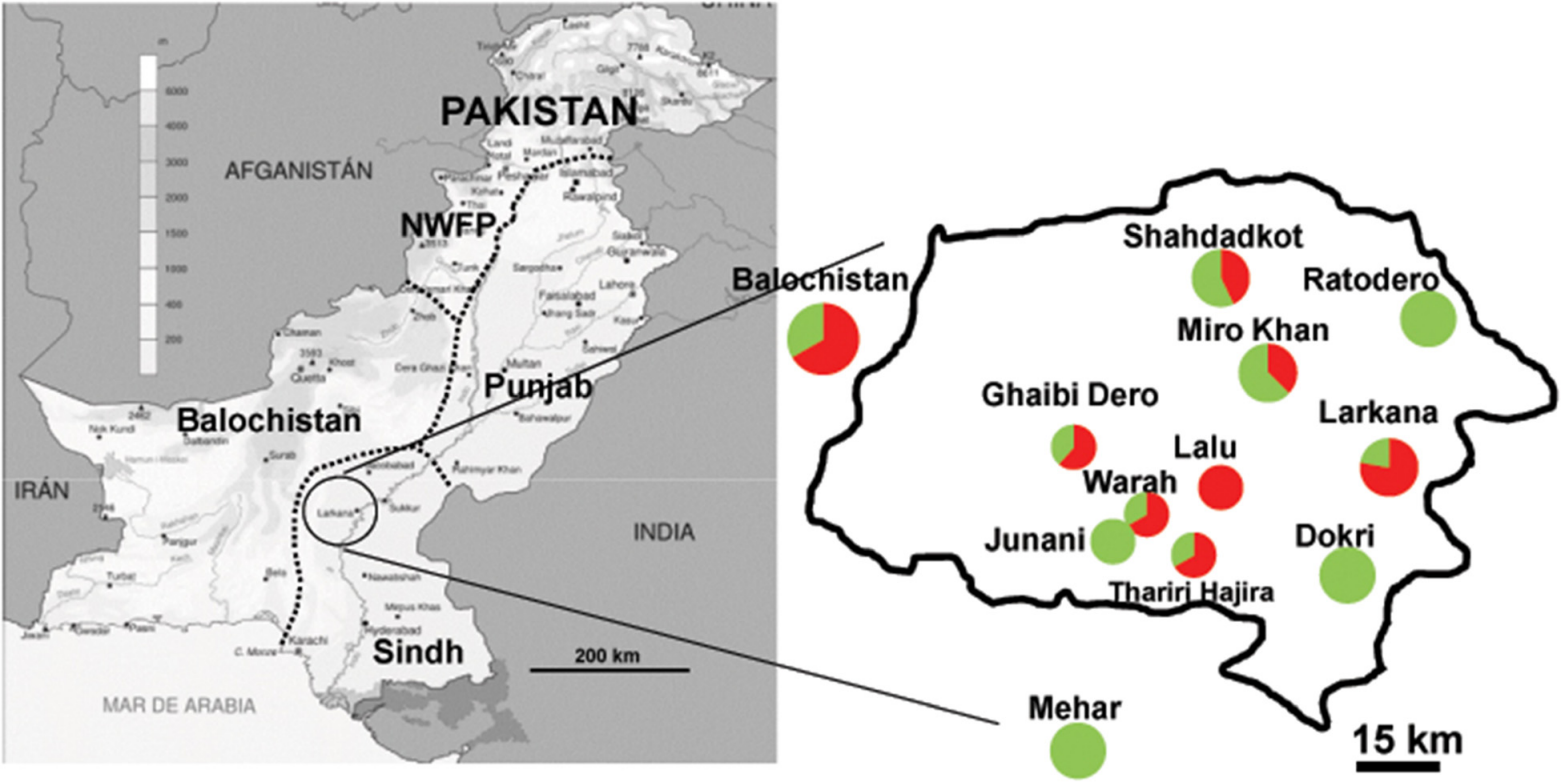
**Map of Pakistan, showing the study area (figure is modified from **[16]**.** Pie charts show the proportion of each population sampled in the respective region. Colours correspond to the population specific ones in Figure 2.

Sixty-six *L. (L.) major* DNA samples isolated from Pakistani CL cases during the period of 2003 to 2004 were analyzed in this study. The patients resided in different villages and cities of Larkana, Shahdadkot and Dadu districts of Sindh province or part of Balochistan province (Figure 1) [15,16]. For 64 samples, the genomic DNA was extracted from amastigotes in skin biopsy specimens using GenomicPrep™ cell and a tissue DNA Isolation Kit (Amersham Pharmacia Biotech, Piscataway, NJ, USA), according to the manufacturer’s instructions [15]. Furthermore, for two strains previously identified as *L. (L.) major* based on parasite-specific kinetoplast DNA (kDNA) sequences [15] the DNA was isolated from cultured promastigotes by using a phenol-chloroform extraction method described previously [17] with some modifications. The source, designation and geographic origin of the parasites from Pakistan analysed in this study are listed in Table 1.

**Table 1:** **Samples of *L. (L.) major *** **analyzed in this study and their multilocus microsatellite (MLMT) profiles**

**Genotype**	**Population**	***L.(L.) major *** **samples**	**Origin**	**4GTG**	**27GTG**	**36GTG**	**39GTG**	**45GTG**	**1GC**	**28AT**	**71AT**	**1GACA**	**1CA**
1	POP-A	P01-43	Ghaibi Dero	7	9	8	2	9	8	18	11	6	18
2	POP-A	P01-44	Unknown	7	9	9	2	9	8	10	11	6	19
3	POP-B	P01-45	Miro Khan	7	9	8	2	9	8	10	12	6	19
4	POP-A	P01-46	Shahdadkot	7	9	8	2	9	8	10	11	6	18
5	POP-B	P01-48	Ghaibi Dero	7	9	8	2	9	8	18	12	6	19
6	POP-A	P01-49	Ghaibi Dero	7	9	9	3	9	8	18	11	6	19
7	POP-B	P01-50	Miro Khan	7	9	9	2	9	8	..	12	6	19
4	POP-A	P01-51	Larkana	7	9	8	2	9	8	10	11	6	18
8	POP-A	P01-52	Warah	7	9	9	2	9	8	18	11	6	19
9	POP-A	P01-59	Ghaibi Dero	7	9	9	2	9	8	10	11	6	18
8	POP-A	P01-61	Miro Khan	7	9	9	2	9	8	18	11	6	19
10	POP-B	P01-62	Junani	7	9	9	2,3	9	8	8	12	6	18
11	POP-A	P01-64	Larkana	7	9	9	2	9	8	10	11	6	20
2	POP-A	P01-66	Lalu	7	9	9	2	9	8	10	11	6	19
12	POP-B	P01-68	Mehar	7	9	8	2	9	8	8	12	6	18
13	POP-B	P02-01	Mehar	7	9	8	2	9	8	10	12	6	20
9	POP-A	P02-02	Balochistan	7	9	9	2	9	8	10	11	6	18
14	POP-B	P02-05	Thariri Hajira	7	8,9	8	2	18	8	18	12	6	..
15	POP-A	P02-10	Shahdadkot	7	9	9	2	9	8	18	11	6	19
16	POP-A	P02-13	Shahdadkot	7	9	7,8	2	9	8	10	11	6	21
17	POP-B	P02-14	Unknown	7	8,9	8	2	9	8	18	12	6	18
18	POP-A	P02-15	Warah	7	9	9	2	9	8	18	11	6	20
19	POP-A	P02-17	Lalu	7	9	8	2	9	8	10	11	6	19
11	POP-A	P02-18	Ghaibi Dero	7	9	9	2	9	8	10	11	6	20
9	POP-A	P02-20	Miro Khan	7	9	9	2	9	8	10	11	6	18
20	POP-B	P02-25	Miro Khan	7	9	9	2	9	8	..	12	6	18
21	POP-B	P02-26	Dokri	7	9	9	2	9	8	8,10	12	6	19
9	POP-A	P02-27	Ghaibi Dero	7	9	9	2	9	8	10	11	6	18
22	POP-B	P02-30	Ratodero	7	9	9	2	9	8	8	12	6	18
23	POP-A	P02-35	Ghaibi Dero	7	8,9	9	2	9	8	10	11	6	18
24	POP-B	P02-36	Dokri	7	9	8	2	18	8	18	12	6	18
9	POP-A	P02-39	Ghaibi Dero	7	9	9	2	9	8	10	11	6	18
25	POP-B	P02-41	Unknown	7	9	8	2	18	8	18	12	6	19
2	POP-A	P02-42	Thariri Hajira	7	9	9	2	9	8	10	11	6	19
26	POP-B	P02-43	Ghaibi Dero	7	9	9	2	9	8	8	12	6	21
27	POP-B	P02-44	Ghaibi Dero	7	9	12	2	18	8	18	12	6	19
28	POP-B	P02-46	Miro Khan	7,10	9	9	2	9	8	8	12	6	21
29	POP-B	P02-54	Warah	7	9	9	2	9	8	10	12	6	18
30	POP-B	P02-55	Dokri	7	9	9,10	2	9	8	8	12	6	19
31	POP-B	P02-65	Shahdadkot	7	9	8	2	9	8	8,10	12	6	20
2	POP-A	P02-66	Balochistan	7	9	9	2	9	8	10	11	6	19
32	POP-B	P02-68	Ghaibi Dero	7	9	8	2	9	8	10	12	6	14
33	POP-B	P02-69	Junani	7	9	12	2	9	8	18	12	6	19
34	POP-B	P02-72	Larkana	7	9	9	2	9	8	8	12	6	20
35	POP-A	P02-79	Miro Khan	7	9	9	2	..	8	18	11	6	18
36	POP-B	P02-86	Dokri	7	9	8	2	18	8	18	12	6	21
19	POP-A	P02-89	Lalu	7	9	8	2	9	8	10	11	6	19
19	POP-A	P02-90	Thariri Hajira	7	9	8	2	9	8	10	11	6	19
22	POP-B	P02-91	Shahdadkot	7	9	9	2	9	8	8	12	6	18
37	POP-A	P02-93	Larkana	7	9	8	2	9	8	18	11	6	21
38	POP-A	P02-94	Larkana	7	9	9	2	9	7	10	11	6	19
39	POP-B	P02-97	Dokri	7	9	8	2	9	8	18	12	6	21
40	POP-B	P02-99	Mehar	7	8,9	8	2	9	8	10	12	6	21
41	POP-A	P02-100	Larkana	7	9	8,10	2	9	8	10	11	6	18
42	POP-B	P02-108	Larkana	7	9	12	2	18	8	18	12	2	19
43	POP-B	P02-109	Shahdadkot	7	9	8	2	9	8	10	12	6	21
44	POP-B	P02-110	Miro Khan	7	8,9	8	2	9	7	10	12	6	18
45	POP-B	P02-119	Ghaibi Dero	7	9	9	2	18	8	18	12	2	18
46	POP-A	P02-123	Unknown	7	9	9	2	9	7	18	11	6	18
47	POP-A	P02-126	Unknown	7	9	9	2	18	8	10	11	6	21
48	POP-A	P02-130	Unknown	7	9	9	2	9	8	18	11	6	18
9	POP-A	P02-131	Larkana	7	9	9	2	9	8	10	11	6	18
27	POP-B	P02-133	Unknown	7	9	12	2	18	8	18	12	6	19
49	POP-A	P02-135	Ghaibi Dero	7	9	9	2	18	8	18	11	6	21
50	POP-A	MHOM/PK/03/SK2*	Larkana	7	9	8	2	9	8	8,9	11	6	14
51	POP-B	MHOM/PK/03/SHD7*	Shahdadkot	7	9	8	2	9	8	8,9	12	6	20

*DNA from cultured parasites was used.

### Microsatellite genotyping

Microsatellite genotyping was carried out using 10 variable microsatellite markers: 4gtg, 27gtg, 36gtg, 39gtg, 45gtg, 1gc, 28at, 71at, 1gaca and 1ca [12]. Fluorescence labeled forward primers were used for the amplification of microsatellite containing sequences applying the PCR condition described previously [12]. The size of the amplicons was determined by capillary electrophoresis with an automated ABI PRISM Gene Mapper sequencer (Applied Biosystem). In each run, a reference strain of *L. (L.) major* (MHOM/IL/1980/Friedlin) was included for which the microsatellite sizes for the 10 loci had been determined by sequencing. MLMType for each strain was obtained by compiling all alleles at each locus. The microsatellite profiles previously described for 130 strains of *L. (L.) major* originated from different geographical areas, including Africa, Central Asia, Iran and Middle East [12,14] were used for comparison.

### Microsatellite data analysis

Multilocus genotype data consists of the number of repeats in each microsatellite markers for each *L. (L.) major* DNA sample analyzed. Population structure was investigated by the STRUCTURE software, which applies a Bayesian model-based clustering approach [18]. This algorithm identifies genetically distinct clusters based on allelic frequencies and estimates the individual’s membership co-efficient in each probabilistic population. A series of 10 runs was performed for each K value between 1 and 10. The following parameters were used: burn in period of 20,000 iterations, 200,000 Markov Chain Monte Carlo iterations, admixture model. The most probable number of clusters was identified as suggested in the software manual by combining the analyses of the mean In Pr (X/K) and the calculation of Δ K, which is based on the rate of change in the log probability of data between successive values of K. The peak of the Δ K graph corresponds to the most probable number of populations in the data set [19].

Microsatellite-based genetic distances were calculated with the software packages MSA [20] and POPULATIONS (http://bioinformatics.org/~tryphon/populations/) by applying the proportion of shared alleles distance measure (Dps). Phylogenetic trees were constructed using Neighbour-joining (NJ) method by the help of the software programmes POPULATIONS 1.2.28 and MEGA [21].

Expected (*H*e, gene diversity) and observed heterozygosity (*H*o) as well as inbreeding co-efficient (*F*is) and mean number of alleles were estimated using the Genetic Data Analysis software (http://hydrodictyon.eeb.uconn.edu/people/plewis/software.php). The degree of genetic differentiation and gene flow among populations were assessed by calculating *F*st values with corresponding *p*-values. *F*st values higher than 0.25 indicate strong genetic differentiation [22].

### Ethical approval

The parasitic DNA were isolated from the human patients’ skin biopsy during the process of laboratory diagnosis of the disease at the outpatient clinic of the Department of Dermatology, Chandka Medical College (a constituent college of Shaheed Mohtarma Benazir Bhutto Medical University), Larkana, Sindh province, Pakistan. The patients were aware that their skin scrapings were needed for diagnosis of the disease using molecular diagnostic methods. Doctors obtained the written consent of the patients. The protocols used were approved by Chandka Medical College, Pakistan.

## Results

Ten polymorphic microsatellite markers were used to analyze 66 samples of *L. (L.) major* collected from CL cases in endemic areas of Sindh and Balochistan province, Pakistan. In total, 51 different multilocus microsatellite profiles summarizing the repeat numbers obtained for the 10 microsatellite markers were assigned to the 66 Pakistani *L. (L.) major* samples tested, of which 43 were unique to individual strains and eight were shared by more than one strain (Table 1). Marker 1CA was the most polymorphic one presenting five alleles, whereas markers 4GTG, 27GTG, 39GTG, 45GTG, 1GC, 71AT and 1GACA were least polymorphic presenting only two alleles for each.

Homozygous allele combinations predominated in the samples studied. Table 2 shows the variability measures of the 10 microsatellite loci, the observed and expected heterozygosities (*H*o and *H*e) as well as the inbreeding co-efficient (*F*is). The *F*is values for 10 markers ranged from −0.0508 to 1. *H*o ranged from 0 to 0.1250 and *H*e ranged from 0 to 0.7488. All markers but one indicated a depletion of heterozygotes. The exception was the 27GTG marker, which revealed an excess of heterozygotes (*H*e < *H*o) corroborated by negative *F*is.

**Table 2:** **Descriptive statistics of the 10 microsatellite markers in the Pakistani *L. (L.) major *** **populations identified by STRUCTURE analysis**

**Locus**	**Population**	**A**	***H***_**e**_	***H***_**o**_	***F*** **is**
4GTG	POP-A	1	0	0	0
	POP-B	2	0.0312	0.0312	0
	Mean	1.5	0.0156	0.0156	0
27GTG	POP-A	2	0.0294	0.0294	0
	POP-B	2	0.1190	0.1250	−0.0508
	Mean	2	0.0742	0.0772	−0.0407
36GTG	POP-A	4	0.4376	0.0588	0.8673
	POP-B	4	0.6145	0.0312	0.9499
	Mean	4	0.5261	0.0450	0.9155
39GTG	POP-A	2	0.0579	0	1
	POP-B	2	0.0312	0.0312	0
	Mean	2	0.0445	0.0156	0.6534
45GTG	POP-A	2	0.1156	0	1
	POP-B	2	0.3809	0	1
	Mean	2	0.2482	0	1
1GC	POP-A	2	0.1123	0	1
	POP-B	2	0.0615	0	1
	Mean	2	0.0869	0	1
28AT	POP-A	4	0.4833	0.0294	0.9400
	POP-B	4	0.6796	0.1000	0.8550
	Mean	4	0.5814	0.0647	0.8903
71AT	POP-A	1	0	0	0
	POP-B	1	0	0	0
	Mean	1	0	0	0
1GACA	POP-A	1	0	0	0
	POP-B	2	0.1190	0	1
	Mean	2	0.0595	0	1
1CA	POP-A	5	0.6935	0	1
	POP-B	5	0.7488	0	1
	Mean	5	0.7212	0	1
Overall	POP-A	2	0.1929	0.0117	0.9398
	POP-B	3	0.2786	0.0318	0.8872
	Mean	3	0.2358	0.0218	0.9088

*A*, the number of alleles per locus; *H*e, the expected heterozygosity; *H*o, the observed heterozygosity; *F*is, the inbreeding coefficient.

**Figure 2:**
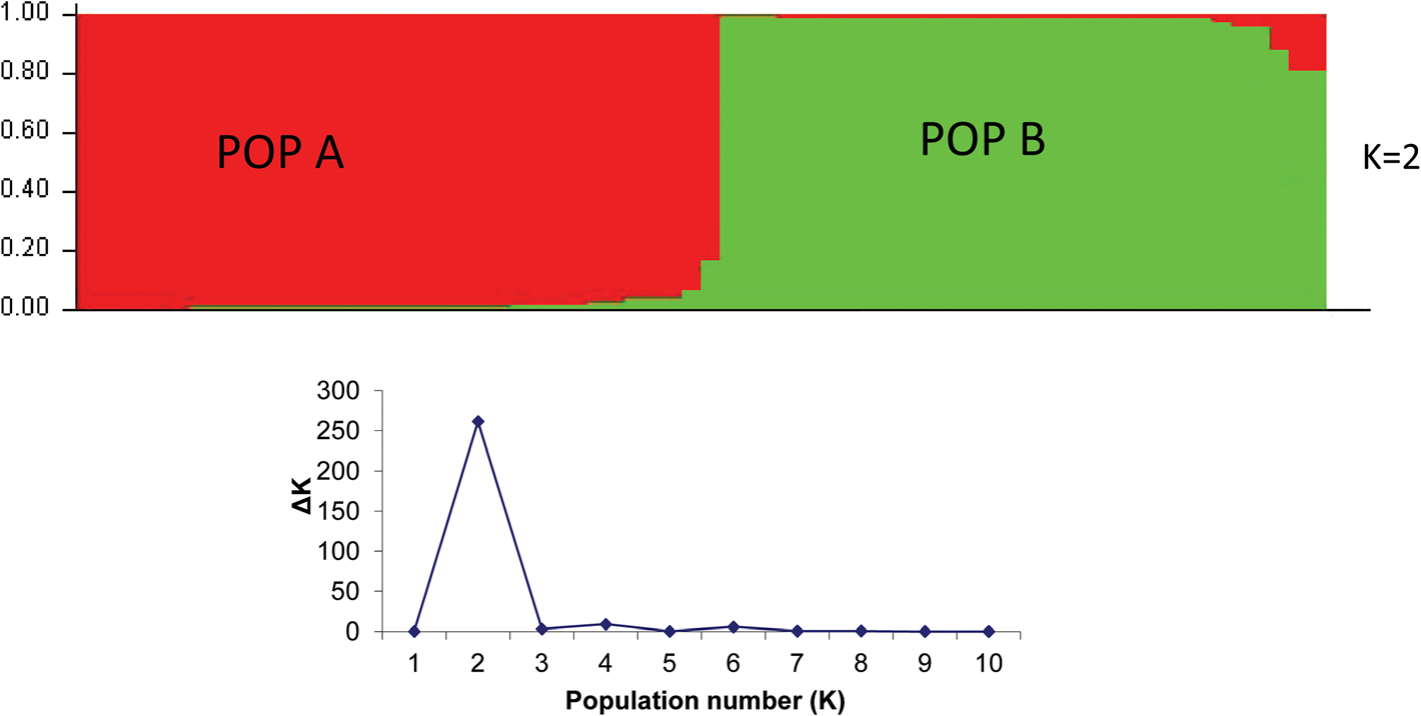
**Estimated population structure for *L. (L.) major *from Pakistan as inferred by STRUCTURE software based on the data for 10 microsatellite markers obtained for the 66** ***L. (L.) major *DNA samples studied herein.** Each strain is represented by a single vertical line divided into K colours, where K is the number of populations assumed. Each colour represents one population, and the length of the colours segment shows the strain’s estimated proportion of membership in that population. The derived graph for ΔK shows at K = 2, indicating the existence of two populations in the investigated strain set.

Bayesian model-based analysis of the 66 samples using STRUCTURE showed that the optimal number of population was 2 (Figure 2). One population (POP-A) consisted of 34 samples; eight from Ghaibi Dero, seven from Larkana, three each from Shahdadkot, Miro Khan and Lalu, two each from Warah, Thariri Hajira and Balochistan, and four of unknown origin. The second population (POP-B) comprised of 32 samples; five each from Ghaibi Dero, Dokri and Miro Khan, four from Shahdadkot, three from Mehar, two each from Larkana and Junani, one each from Warah, Thariri Hajira and Ratodero, and three of unknown origin. These two populations were significantly different as shown by their *F*st value (0.4329) and *P*-value 0.0001.

**Figure 3:**
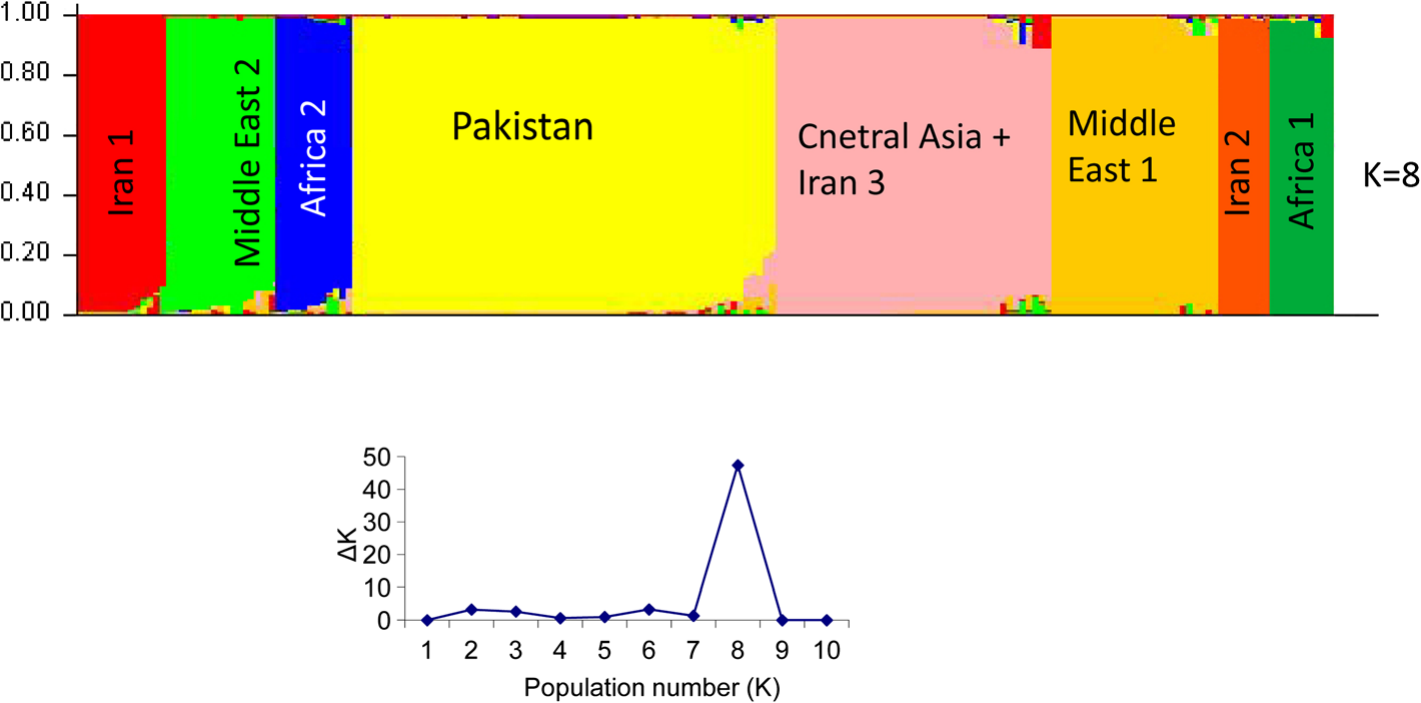
**General population structure estimated for 196 *L. (L.) major *microsatellite profiles, including the 66 from Pakistan, compared in this study.** In the bar plots each strain is represented by a single line divided into K colours, where K is the number of population. Isolates are organized by membership coefficients.

When merging the 66 Pakistani samples with 130 *L. (L.) major* strains from 20 countries in Central Asia, Africa, Iran and Middle East, STRUCTURE analysis assigned these 196 strains to 8 different populations (Figure 3). Seven of these corresponded to the previously exposed populations Central Asia/Iran 3, Middle East 1, Middle East 2, Africa 1, Africa 2, Iran 1, Iran 2 [14]. The eighth population comprised all strains from Pakistan which were found to be clearly distinct from the other strains of *L. (L.) major* studied so far. The Pakistani population was re-analysed separately by STRUCTURE and split into the two sub-populations described above (Figure 2).

**Figure 4:**
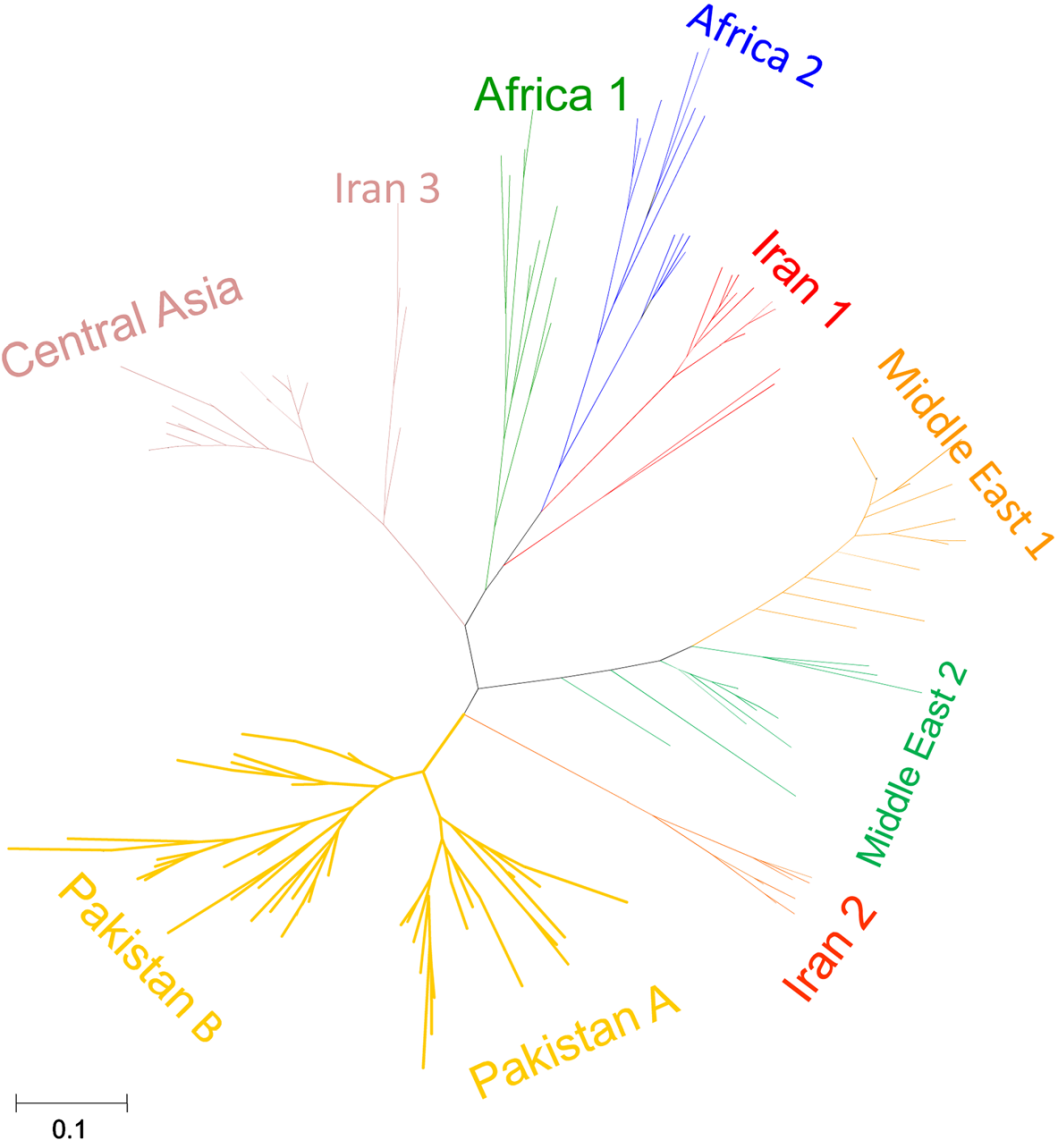
**Neighbour-joining tree (unrooted) inferred from the Dps distances for the 196 *L. (L.) major *microsatellite profiles, including the 66 from Pakistan.**

The unrooted NJ tree constructed for the 196 strains of *L. (L.) major* using MEGA assigned the Pakistani samples to the same two clusters (Figure 4) as STRUCTURE analysis did. *F*-statistics revealed a significant amount of population structure among all of *L. (L.) major* clusters. The *F*st values (Table 3) were significant and higher than 0.25 indicating very great genetic differentiation between the populations.

**Table 3:** ***F*** **st values (upper triangle) and corresponding *P*-values (lower triangle) for the populations and sub-populations of *L. (L.) major *** **as assumed by STRUCTURE**

***F*** **st-values**	**Pakistan A**	**Pakistan B**	**Central Asia/Iran 3**	**Africa 1**	**Africa 2**	**Middle East 1**	**Middle East 2**	**Iran 1**	**Iran 2**
Pakistan POP-A	0	0.3351	0.7336	0.5924	0.6491	0.7483	0.6174	0.7174	0.6354
Pakistan POP-B	0.0001	0	0.6863	0.5326	0.5674	0.7013	0.5508	0.6056	0.5120
Central Asia	0.0001	0.0001	0	0.7066	0.6667	0.7094	0.7646	0.7861	0.7356
Africa 1	0.0001	0.0001	0.0001	0	0.2626	0.5974	0.4807	0.4995	0.5301
Africa 2	0.0001	0.0001	0.0001	0.0001	0	0.6336	0.5774	0.4630	0.5214
Middle East 1	0.0001	0.0001	0.0001	0.0001	0.0001	0	0.62226	0.7819	0.7571
Middle East 2	0.0001	0.0001	0.0001	0.0001	0.0001	0.0001	0	0.6704	0.6561
Iran 1	0.0001	0.0001	0.0001	0.0001	0.0001	0.0001	0.0001	0	0.6592
Iran 2	0.0001	0.0001	0.0001	0.0001	0.0001	0.0001	0.0001	0.0001	0

*F*st-value: 0–0.05 (little genetic differentiation), 0.05-0.15 (moderate genetic differentiation), 0.15-0.25 (great genetic differentiation), above 0.25 (very great genetic differentiation).

## Discussion

In this study, the diversity and population genetic structure of strains of *L. (L.) major* from Pakistan was investigated, compared, and correlated with their geographical sources and prevailing environmental and ecological conditions. The present MLMT analysis revealed considerable genetic variation for the 66 Pakistani *L. (L.) major* DNA samples presenting 43 individual microsatellite profiles and eight were shared by several samples. This is a quite unexpected result because all the samples studied were from different villages and cities of Larkana, Shahdadkot and Dadu districts of Sindh province, except three that came from Balochistan province. Heterogeneity of Pakistani *L. (L.)major* is thus much higher as previously suggested when little intra-specific polymorphism was found for the parasites from the same area [6]. According to *F*is, *H*o and *H*e values, microsatellite loci were mostly homozygous in the Pakistani sample set. *Leishmania* species have been considered to be clonal diploid organisms [23] in which *F*is values are supposed to be negative due to heterozygote accumulation [24]. In this study, significant heterozygote deficiency was observed for most of the microsatellite loci. Heterozygote deficiency could result from population subdivision (Wahlund effect), presence of null alleles, natural selection, genetic conversion and inbreeding as discussed by Rougeron *et al*. (2009) [25]. In our study, almost all *L. (L.) major* DNA isolates came from the same area. Thus, the heterozygote deficiency found in the studied samples is unlikely to be due to the Wahlund effect (geographical isolation). In our study, 62 strains were amplified at all microsatellite loci and only four strains had one missing locus each (ca. o.6% of all loci), but our data analysis using Micro-Checker software (http://www.microchecker.hull.ac.uk/) showed evidence for a null allele with few microsatellite loci (45GTG, 28AT and 1CA). Therefore, we cannot exclude the presence of heterozygote deficiency could result from null alleles. The high *F*_IS_ values observed across all polymorphic loci are also likely to be due to inbreeding. Selection may cause under-dominance by decreasing the fitness of heterozygous genotypes and gene conversion could lead to a transition from heterozygous to the homozygous stage [25]. In both cases, varying *F*_IS_ should be expected across our 10 non-coding microsatellite loci. As can be seen in Table 2, in locus 71 AT the expected and observed heterozygosities were zero for both POP-A and POP-B, and in locus 1GACA this was the case for POP-A. In locus 27GTG expected and observed heterozygosities were almost equal for both populations and the same was observed for POP-B in loci 4GTG and 39GTG. The resulting *F*_IS_ values were zero or close to zero suggesting that these loci are in equilibrium. All other loci had high *F*_IS_ values indicating heterozygote deficiencies. Due to the variance of *F*is values across the microsatellite loci, we cannot exclude that selection or gene conversions have contributed to the high *F*_IS_ values observed in this study. We would, however favor the hypothesis of significant inbreeding present in both Pakistani populations of *L. major*, as previously reported for different *Leishmania* parasites [25-27].

The Bayesian clustering approach implemented in STRUCTURE as well as the phylogenetic analysis based on genetic distances assigned the 66 Pakistani *L. (L.) major* samples to two populations (POP-A and POP-B). *F*statistics confirmed that these are genetically isolated populations. The two samples from Balochistan belonged to Population A. The Pakistani populations identified in the present study where clearly separated from the populations comprising of *L. (L.) major* strains from Central Asia, Africa, Iran and Middle East.

The two Pakistani populations did not correlate with the geographical origin of the parasites that fell into them. Their analysis was, however, hampered owing to the small number (only 2) of DNA samples available from Balochistan province. The geographical overlap between two genetically isolated populations might be due to introduction of parasites from different foci through human or reservoir migrations and vector sandfly habitat expansion. One of the most important risk factors in the increase of CL worldwide has been the migration of people from endemic regions [28]. The occurrence of different eco-epidemiological situations, different sand fly vectors and different reservoir hosts might be another explanation for the co-existence of two distinct populations in the same geographical area. Two sand fly species, *Phlebotomus papatasi and P. salehi,* and three rodent species (*Meriones hurrianae, Rhombomys opimus,* and *Tatera indica*) are incriminated as vectors and reservoirs, respectively, of *L. (L.) major* parasites in Pakistan [1]. It is assumed, that *L. (L.) major* in Sindh province, Pakistan has distinct epidemiological and biological characteristics. Variations among the samples of *L. (L.) major* from the same endemic area leading to assignment to different populations were previously attributed to differences in sand fly vector populations [29] and reservoir hosts [30]. Indeed, the existence of distinct groups of Pakistani *L. (L.) major* suggests that the extant parasites in Pakistan may have been restricted there for a long time, rather than being recently introduced from elsewhere by human or animal reservoir migration. The same scenario was recently obseved for *L. (L.) tropica* in Morocco [31] where two genetically very distinct co-existing populations within the same focus were identified. Pratlong *et al*. (1991) [32] speculated that this old focus was colonized by strains of different geographical origins and that these strains diversified into lesser variants apparently by recent mutation. As there is no epidemiological information available about the strains studied herein it is not possible to judge what the underlying reason(s)/factor(s) for the existence of two genetically distinct populations of *L. (L.) major* in Sindh province, Pakistan, is.

Our study demonstrated the possibility and usefulness of performing MLMT using skin biopsy materials from patient tissues that contain only small amounts of *Leishmania* DNA. We succeeded in amplifying 10 microsatellite loci from 64 clinical DNA samples. Parasite culture is not easy to perform, especially under field conditions, and often not successful. Therefore, assays that can be carried out directly on clinical materials are of great advantage for surveys including high numbers of isolates. In addition, the direct DNA isolation of *Leishmania* from clinical samples would avoid the potential selection of special parasites during *in vitro* cultivation.

## Conclusions

To the best of our knowledge, this study is the first one that has investigated the population structure and genetic diversity of *L. (L.) major* in Pakistan by using the MLMT approach. We were able to detect two genetically isolated populations of *L. (L.) major* in Sindh province, Pakistan. Furthermore, our results corroborated the possibility and/or usefulness of genotyping *L. (L.) major* directly from clinical samples [33,34]. A comprehensive study of the epidemiology of CL in Pakistan, including more strains from other regions endemic for CL and investigations of possible differences in reservoirs and sand fly vectors, is warranted.

## Competing interests

The authors declare that they have no competing interests.

## Authors’ contributions

MZA designed the study, performed microsatellite typing, conducted data analysis and wrote the manuscript. AMB, FRS, JHB, HK, HU and YH collected the skin biopsy samples and isolated the DNA. RN conducted data analysis. GS participated in coordination and helped to draft the manuscript. YH, HK and KK conceived of the study and critically revised the manuscript. All authors have read and approved the final manuscript.
